# Twitter as a Mental Health Support System for Students and Professionals in the Medical Field

**DOI:** 10.2196/17598

**Published:** 2021-01-19

**Authors:** Lisa Liu, Benjamin K P Woo

**Affiliations:** 1 Department of Molecular, Cell, and Developmental Biology University of California, Los Angeles Los Angeles, CA United States; 2 Department of Psychiatry University of California, Los Angeles Los Angeles, CA United States

**Keywords:** Twitter, social media, mental health, health professionals, community, social support, depression, physician suicide

## Abstract

Twitter is a rapidly growing social media site that has greatly integrated itself in the lives of students and professionals in the medical field. While Twitter has been found to be very helpful in facilitating education, there is also great potential for its usage as a social support system. Social support has become more essential as society grapples with declining mental health, particularly in the medical sector. In our previous paper, we saw that Twitter provides a promising tool to learn more about the online conversation about dementia and, in particular, the supportive network that can be created. Inspired by this, we decided to investigate the potential of using Twitter as a support system for students and professionals in the medical field. In this paper, we explore the current state of mental health in the medical field and suggest practical implementation methods for using Twitter.

## Introduction to Twitter

Twitter is a free social networking and microblogging site that was launched in 2006. Users can send out brief, 280-character messages to either the public or to a specific subset of approved followers. These messages can then be retweeted and shared with another user’s followers, which can lead to a ripple effect, with messages spreading to larger social networks. Upon seeing the tweets, others can immediately respond, allowing for almost instantaneous dialogue. Messages can include a hashtag (#) for specific words or phrases, allowing users’ messages to be searchable.

In the past decade, Twitter has been used more frequently by the medical community [1-3]. A 2011 research letter published in *JAMA* describes how physicians frequently use Twitter to share medical information and discuss health topics [4]. A review from 2017 found that social media, including Twitter, can be a useful tool to be supplemented with the medical curriculum [5]. Another paper by Jayaram et al [6] noted that Twitter can be helpful for the medical community, as it provides a useful platform during conferences by fostering discussion and sharing content. Interestingly, a study published in 2018 investigating the use of the #TipsForNewDocs hashtag found that over the course of 2 days, 661 unique posts containing this hashtag were posted by doctors, health care professionals, and patients. While most of the tweets were focused on improving personal or professional qualities, there was a significant number of tweets on socialization and creating a welcoming community [7]. While Twitter can greatly facilitate academic discussion among health professionals and students, Twitter can also provide a supportive community for these individuals by fostering a sense of community and allowing individuals to support each other through tweets, likes, and comments. In this paper, we investigate the current state of mental health in the medical field and suggest some practical implementations of using Twitter.

## Mental Health Concerns in the Health Profession

Mental health is a rising concern for individuals in the health profession. One study using data from 43 countries found that 27.2% of medical students showed depression symptoms, while only 15.7% sought treatment [8]. Similarly, it has been found that 20% of medical residents had symptoms of depression, with 74% of them satisfying criteria for burnout [9,10]. These concerns are detrimental not only for the students themselves but also for their patients, as it was found that residents with depression were 6.2 times more likely to make medication errors than nondepressed residents [10]. A study from 2004 showed that the suicide rates among male and female physicians are higher than those of the general population [11]. Finally, in a 2014 article, Dr Sinha [12] brought to light the issues of physician suicide and revealed his own experiences with the stresses of the medical profession. Several papers note that there may be negative consequences for physicians who acknowledge their mental health problems, as they may lose their medical license or the opportunity for career advancement [13-15]. If physicians are afraid to speak out or seek help, the problem may become worse. Thus, there is a great need for increased platforms to safely share these personal experiences and difficulties in a supportive community, especially in a way that can normalize mental health issues. Twitter provides a promising technological tool for this.

## Value of a Supportive Online Community

A supportive online community can play a large role in improving health by helping to reduce feelings of social isolation. Although excessive social media use can be detrimental to mental health and getting adequate sleep [16], a limited amount of social media use, in which one engages in a supportive network with shared experiences, can be beneficial. Using technology for such social interactions removes limitations of geography, time zones, work schedules, and illnesses [17]. Online platforms can promote individuals to share health information and advice and encourage one another to adhere to recommended lifestyle changes [17]. It has even been found that people with serious mental illness report benefits from interacting with peers online due to greater social connectedness and feelings of group belonging through connecting through personal stories and coping strategies [18]. Although there are many types of social support (ie, informational, emotional, instrumental), this paper focuses on the emotional support that social media can provide.

Research has shown that Twitter can provide valuable psychological support. Our previous paper showed that Twitter has potential to create a valuable social support network for individuals affected by dementia and their family members [19]. Meanwhile, using the hashtag #WhyWeTweetMH, Berry et al [20] found that most people tweeted about mental health because of the sense of community, to raise awareness and combat stigma, and to have a safe space for expression and empowerment, creating a potential therapeutic effect.

Finally, a study by Sugawara and colleagues [3] showed that cancer patients can empower themselves by tweeting information about their own medical condition and treatment, providing a valuable forum for open discussion. After a thorough selection process described in the paper, the researchers focused on the account from one female cancer patient. The researchers noticed a majority of the tweets were related to psychological encouragement. User 1 wrote that she had “cleared the blood test,” and this was followed by the comment “Glad to hear that you cleared the test!” from user 2. In this scenario, Twitter provided a space for an individual to share what she was doing and to receive encouragement from the online community.

As the papers by Berry et al [20] and Sugawara et al [3] show, Twitter can provide a supportive online community that allows individuals to write about their experiences or feelings and to receive positive or encouraging feedback.

## Implications for Individuals in the Medical Field

Implementing a hashtag to discuss a particular topic, as was done in Berry et al [20] (#WhyWeTweetMH) and Hennessy et al [21] (#nlm2soton), can allow for the aggregation of relevant content in this open, searchable space that is the online community of Twitter. Similarly, for physicians, perhaps a hashtag such as #AnesthesiologistStruggles can be used to share stresses that they face on a daily basis and discuss coping methods.

Using specific hashtags would allow us to congregate information on a particular topic to see how people are feeling about that topic. Are they stressed out about an upcoming exam? Are they struggling with a procedure? One study on #colorectalsurgery provides an account of how a community of colorectal surgeons were able to come together to share experiences on surgical techniques [22]. Analyzing such topics of conversation can allow individuals in the medical field to come up with solutions to help solve some of these problems. Perhaps noticing an online community of colorectal surgeons struggling with a particular technique will encourage clinicians to rethink the way that technique is performed and realize it is okay to openly share their struggles or inspire a colorectal surgeon who sees that post to recognize that this is a common problem and to work toward a solution to help.

Posting about personal struggles will allow stigmas to become more normalized. If individuals read many posts about a topic, such as struggling with mental health, from people they know, it seems more normalized because they may think, “So many of my friends and classmates are going through these struggles, perhaps it is something common.”

One method of creating such a forum is described in Admon et al [23]. Their strategies for organizing a Twitter chat include (1) thinking about the purpose of creating a Twitter chat, (2) identifying appropriate moderators, and (3) effectively publicizing the chat [23].

It would be beneficial for these conversations to be regulated in order to limit irrelevant material, misleading or false information [18,24], derogatory comments from others [18], and accidental violation of patient privacy [4,24]. A paper by Hennessy et al [25] provides an overview of some guidelines for health professionals to follow when delving into the social media community. Although Hennessy et al [25] places an emphasis on using social media to interact with patients, which is not the focus of this paper, several of the guidelines presented are applicable in this case, such as (1) making sure that patient confidentiality does not get breached in any way; (2) being respectful of other people’s ideas, opinions, and habits; and (3) not posting copyrighted material as the user’s own [25].

## Conclusion

Declining mental health in an important issue that students and professionals in the medical field face. Social media platforms, such as Twitter, provide a promising space for individuals to find a supportive community, share experiences, and receive helpful advice. Previous research on Twitter has found supportive communities for individuals affected by dementia, mental health, and cancer. Thus, Twitter could also provide a community for improving mental health for individuals specifically in the medical field. Using specific hashtags may be useful for facilitating such communities.

Online social networks have the potential to extend social circles at the cost of in-person social interactions, resulting in increased social isolation [17,26]. With this in mind, it is important to remember that social support provided by Twitter is not meant to replace valuable human interaction but rather to provide a convenient supplemental tool.
